# Intravesical Instillations of Hyaluronic Acid as First-Line Treatment in Patients with Interstitial Cystitis/Bladder Pain Syndrome: Use, Efficacy and Effects on Quality of Life

**DOI:** 10.3390/healthcare12121190

**Published:** 2024-06-13

**Authors:** Francesco Plotti, Gianna Barbara Cundari, Fernando Ficarola, Corrado Terranova, Carlo De Cicco Nardone, Roberto Montera, Daniela Luvero, Federica Guzzo, Adele Silvagni, Amerigo Ferrari, Donatella Caserta, Roberto Angioli

**Affiliations:** 1 Research Unit of Gynaecology, Fondazione Policlinico Universitario Campus Bio-Medico, Via Alvaro del Portillo 200, 00128 Rome, Italy; f.plotti@policlinicocampus.it (F.P.); ficarola.fernando@gmail.com (F.F.); c.terranova@policlinicocampus.it (C.T.); c.decicconardone@policlinicocampus.it (C.D.C.N.); r.montera@policlinicocampus.it (R.M.); d.luvero@policlinicocampus.it (D.L.); f.guzzo@policlinicocampus.it (F.G.); adele.silvagni@unicampus.it (A.S.); r.angioli@policlinicocampus.it (R.A.); 2 Research Unit of Gynaecology, Department of Medicine and Surgery, Università Campus Bio-Medico Di Roma, Via Alvaro del Portillo 21, 00128 Rome, Italy; 3 Division of Obstetrics and Gynaecology, Department of Clinical and Experimental Medicine, University of Pisa, 56126 Pisa, Italy; amerigo.ferrari@santannapisa.it; 4 Department of Surgical and Medical Sciences and Translational Medicine, Sapienza University of Rome, Sant’Andrea Hospital, Via di Grottarossa, n. 1035/1039, 00189 Rome, Italy; donatella.caserta@uniroma1.it

**Keywords:** interstitial cystitis, bladder pain syndrome, hyaluronic acid, glycosaminoglycans

## Abstract

The efficacy of hyaluronic acid instillations as therapy for patients with Interstitial Cystitis/Bladder Pain Syndrome (IC/BPS) has been demonstrated in some clinical studies, with response rates up to 70%. The aim of the study is to investigate the change in symptoms and quality of life in female patients with IC/BPS after intravesical instillations of hyaluronic acid used as first-line treatment. A retrospective single-center cohort study was conducted. Female patients, whose symptoms were compatible with the diagnosis of IC/BPS as defined by the International Continence Society, were treated with a variable number of intravesical instillations of a hyaluronic acid-based drug. Three validated questionnaires were administered by telephone to all patients, before the beginning of the treatment and 6 months after the last administration of the drug. A total of 50 patients with symptoms compatible with the diagnosis of IC/BPS were included in the study. The median number of instillations performed is 4. For all questionnaires, the median value was significantly reduced following treatment with intravesical instillations (*p* = 0.000). The present study has shown that intravesical hyaluronic acid treatment results in both statistically and clinically significant symptomatic improvement, thereby improving the quality of life of patients with IC/BPS.

## 1. Introduction

Interstitial cystitis, a chronic inflammatory condition of the bladder, has been defined variably throughout the years, reflecting evolving understandings of its pathology. The definition of interstitial cystitis has largely changed over the years until 2017 when the definition of this condition was merged with the definition of Bladder Pain Syndrome. Thus, the two nosological entities have been unified. In fact, as defined by the International Continence Society (ICS), Interstitial Cystitis/Bladder Pain Syndrome (IC/BPS) is “Persistent or recurrent chronic pelvic pain, pressure or discomfort perceived to be related to the urinary bladder accompanied by at least one other urinary symptom such as an urgent need to void or urinary frequency” [1].

Numerous epidemiological studies have been conducted worldwide to identify the correct prevalence and incidence of IC/BPS; however, so far, they have all highlighted several problems. The lack of a universally accepted definition, the absence of a validated diagnostic method and the still open questions regarding the etiology and pathophysiology of the disease make a consistent part of the literature difficult to interpret. However, current evidence estimates a worldwide prevalence rate ranging from 0.01% to 6.5% [2]. The prevalence of the condition ranges from 52 to 500/100,000 in females compared to a range of 8–41/100,000 in males, and its incidence has been conservatively estimated at 1.2/100,000 [3].

IC/BPS is characterized by a loss of integrity of the bladder urothelium, which allows irritants and bacteria to penetrate the underlying tissue, causing an inflammatory process. This could result from reduced production or damage of glycosaminoglycans (GAGs), which form the initial protective barrier of the bladder wall [4]. The urothelium is covered by a GAG layer, whose natural constituents include hyaluronic acid, a non-sulfated GAG, and the sulfated GAGs chondroitin sulfate, heparan sulfate/heparin keratan sulfate, and dermatane sulfate [5]. Despite various research attempts, there are no diagnostic clinical markers of the disease. Patients showing symptoms attributable to IC/BPS are screened to exclude gynecological or urological pathologies of bladder and/or urethral competence or pathologies belonging to the musculoskeletal system. Hence, the diagnosis of IC/BPS remains a diagnosis of exclusion [6].

The impossibility of establishing a standardized treatment is attributable to the poor pathophysiological understanding of the disease. Several hypotheses have been formulated on the origin of the symptoms of IC/BPS, each considering a different etiology. As a result, it is not possible to identify a universally recognized therapeutic pathway. Therefore, the aim of therapy is to manage symptoms and improve patients’ quality of life through individualized targeted therapy [7].

There are numerous therapeutic strategies available for IC/BPS. Specifically, recommendations published by the American Urological Association (AUA) suggest as first-line therapies: general relaxation or stress management, pain management, patient education, and self-care/behavioral management [8]. Subsequently, oral therapies with amitriptyline, cimetidine, Pentosan Polysulfate (PPS) and hydroxyzine, or intravesical therapies with dimethyl sulfoxide (DMSO), heparin, or lidocaine, are proposed as subsequent treatment strategies [8,9]. Third and fourth-line therapies according to AUA include cystoscopy with hydrodistension, intradetrusor injection of botulinum toxin A (BTX-A), and eventually neuromodulation [8,10]. New and interesting therapeutic strategies involve combined approaches that include dietary modifications along with botulinum toxin injections or intravesical instillations of hyaluronic acid [11]. Additionally, the use of immunomodulatory compounds represents a new area of interest [11,12,13].

Among the numerous therapies proposed for IC/BPS, intravesical ones are particularly effective; this is due to the administration of drugs in high concentrations directly within the bladder, achieving better absorption of the drug with improved bioavailability, thereby decreasing side effects [14,15]. Based on the hypothesis that damage to the GAG layer is a cause of symptoms in IC/BPS, intravesical GAG replenishment therapy is widely used to treat these patients [5]. The efficacy of hyaluronic acid instillations as therapy for patients with interstitial cystitis has been demonstrated in some clinical studies, with response rates up to 70% [16,17,18]. However, there is a lack of consistent data in the literature on this topic and most of the studies currently available present uneven data and small samples.

The primary outcome of this study is to investigate the change in symptoms and quality of life in female patients with IC/BPS after intravesical instillations of hyaluronic acid used as first-line treatment. The secondary outcome is to assess whether the number of intravesical instillations of hyaluronic acid influences the clinical outcome of the disease in a statistically significant way.

## 2. Materials and Methods

### 2.1. Study Design

We conducted a detailed retrospective cohort study at a single center. After consulting with our local Ethical Committee, our study was classified as exempt from Internal Review Board oversight, because it was observational and non-interventional (no randomization occurred), reflecting the retrospective design of the study. This study was conducted following the regulatory standards of Good Clinical Practice and the Declaration of Helsinki. The clinical management of patients included in the study was identical to that routinely proposed in the same period according to our internal protocols. For this reason, all eligible patients were thoroughly informed about the nature and objectives of the study, and written consent was obtained in compliance with the Italian Privacy Law (675/96).

### 2.2. Study Population

The sample examined included female patients of any age, admitted to Campus Bio-Medico University Hospital Foundation in Rome, presenting symptoms compatible with the diagnosis of IC/BPS, according to the validated definition of the ICS [1]. All patients reported chronic pelvic pain, a sense of pressure or bladder discomfort associated with at least one urinary symptom between urinary urgency and pollakiuria. We selected all patients who had performed urinalysis, urine culture, diagnostic cystoscopy with biopsy, and invasive urodynamic tests prior to treatment. All patients included in the study had symptoms compatible with the diagnosis of IC/BPS, negative urinalysis and urine culture, histological outcome of the biopsy performed during the diagnostic cystoscopy of chronic inflammation with or without Hunner lesions and negative invasive urodynamic tests. All patients included in the study had no previous therapy for IC/BPS. The study excluded all patients with positive urinalysis and urine culture, diagnostic cystoscopy with negative biopsy for chronic inflammation, diagnosis by invasive urodynamic tests of urinary incontinence, contraindications to intravesical instillations, hypersensitivity or allergy to one of the components of the drug, bladder endometriosis, anterior compartment prolapse and/or central compartment prolapse grade ≥ 3 according to Baden and Walker classification, ongoing vaginal infections, diagnosis of pudendal nerve neuralgia, patients with neoplastic diseases of the genitourinary tract, continuous use of diuretic drugs, pregnant or breastfeeding patients, previous pelvic surgery and diagnosed neurological disorders.

### 2.3. Study Procedures

All patients included in the study received a varying number of intravesical instillations of a hyaluronic acid-based drug. All patients included in the study were offered a minimum number of 4 intravesical instillations. Patients who performed a minimum number of 2 intravesical instillations were included in the study. No upper limit in the number of intravesical instillations performed was considered. Each instillation was performed at a minimum distance of 30 days from the previous one. The drug used for the instillations is composed of a sterile solution made of water, calcium chloride (0.87%—440 mg/50 mL), hyaluronic acid (1.6%—800 mg/50 mL) and sodium chondroitin sulphate (2%—1 g/50 mL). All contents are enclosed in pre-filled 50 mL syringes injected into the bladder through a bladder catheter during an outpatient visit. Before starting the procedure, the patient is asked to urinate; complete bladder emptying is then ensured with the use of an extemporaneous bladder catheterization. To consider the treatment valid, the patient must hold the drug in the bladder for at least 1 h. All patients meeting the inclusion criteria provided informed consent before participating in the study, which involved administering anonymous questionnaires via telephone. For ethical reasons, privacy and data confidentiality is guaranteed for all patients in this study.

As primary outcome, validated questionnaires were administered via telephone to all patients included in the study, to assess symptoms both before the start of treatment and six months following the final drug administration. The questionnaires used were the following three: the O’Leary’s Interstitial Cystitis Symptoms Index (ICSI), the O’Leary’s Interstitial Cystitis Problem Index (ICPI) [19] and the Visual Analogue Scale (VAS). The ICSI is a validated questionnaire containing four questions defining, in order, urinary urgency, frequency of urination, nocturia and bladder pain/burning sensation. For each question, patients are requested to assign a numerical value ranging from 0 to 5, where 0 indicates the complete absence of the symptom and 5 indicates the symptom’s very frequent occurrence. Within the response range, values from 0 to 2 indicate a low level of symptomatology, from 3 to 5 a high level. The ICPI is a validated questionnaire consisting of four questions, which measures the impact of the symptoms described in ICSI on patients’ quality of life. For each question, patients are requested to assign a numerical value ranging from 0 to 4, where 0 signifies the least discomfort and 4 indicates the most discomfort. Answers falling in the range from 0 to 2 indicate medium to low discomfort; responses falling in the range from 3 to 4 indicate high discomfort derived from the symptom investigated. The VAS defines a visual representation of the intensity of the pain felt by the patient which is translated into a number from 0 to 10, where 0 indicates the absence of pain and 10 indicates the maximum pain perceived by the patient. This scale has been used to define chronic pelvic pain, pressure or bladder discomfort perceived by the patient.

As a secondary outcome, we assessed the post-treatment differences in the ICSI, ICPI and VAS scores between women who underwent a high number of sessions (>4) and those who underwent a low number of sessions (≤4). The threshold of 4 sessions as the upper limit for defining a low number of sessions was established based on the median number of sessions undertaken by the patients included in the study.

### 2.4. Statistical Analysis

A descriptive statistical analysis was conducted for all the parameters listed above; specifically, the median and Interquartile Range (IQR) were calculated for the continuous variables, while the absolute frequencies and percentages were calculated for the categorical variables. The statistical analysis was conducted by comparing the scores recorded before and after treatment, by means of Student’s *t*-test for paired data and the corresponding non-parametric test and of Wilcoxon’s test for paired data. We then examined whether a statistically significant mean difference existed between the three pre-treatment and post-treatment scores. We also assessed by using the chi-square test whether post-treatment scores varied between women performing a high number of sessions (>4) and women performing a low number of sessions (≤4). A p value of less than 0.05 was considered to indicate statistical significance. The sample size was calculated in terms of the difference between treatments on VAS pain level from baseline to 6 months considering a mean effect reduction of 1.8 (which is the minimally important clinical difference according to the literature). Considering a VAS score before treatment of 7.6 ± 3.3, a mean reduction of 1.8, a power of 95%, an α level of 0.05, and a 15% dropout rate, a total of 51 patients were considered enough to maintain the statistical power of the study.

## 3. Results

A total of 55 patients aged between 17 and 85 years, presenting symptoms compatible with the diagnosis of IC/BPS, were recruited between November 2022 and March 2023. Of these, five patients were excluded due to non-compliance with the inclusion criteria: three had positive urine cultures and two were diagnosed with urinary incontinence following a urodynamic testing. Consequently, 50 patients who received between 2 and 15 intravesical instillations of a hyaluronic acid-based drug were included in the study. A total of 19 patients received fewer than four sessions (the minimum number recommended by the gynecologist during the outpatient visit) because they reported satisfactory improvement in symptoms and chose not to continue treatment.

Table 1 shows the descriptive characteristics of the 50 patients included in the study.

The median age of the participants was 53.5. The median number of instillations administered was four. Of these patients, 31 (62%) received four or fewer instillations, while the remaining 19 (38%) underwent more than four sessions.

In Table 2, we report the median and the IQR for the scores of the three questionnaires administered before treatment and 6 months afterward.

For all three measures, there was a significant reduction in the median scores following treatment with intravesical instillations.

To determine whether the differences between pre-treatment and post-treatment scores were statistically significant, a Student’s *t*-test for paired samples was conducted for each score (ICSI, ICPI and VAS), with the results displayed in Table 3.

The analysis shows that the differences were statistically significant (*p* = 0.000) for all three scores. To verify the robustness of these findings, the same calculations were repeated using the non-parametric counterpart of Student’s *t*-test, i.e., Wilcoxon’s test for paired data, which confirmed the initial results.

As shown in Table 4, the mean differences in post-treatment scores for all three assessments—ICSI, ICPI, and VAS—between patients who underwent a low number (≤4) of sessions and those who underwent a high number of sessions did not reach statistical significance (*p* > 0.05).

This suggested that the number of intravesical instillations does not significantly impact the clinical outcomes for the patients.

## 4. Discussion

IC/BPS is an extremely disabling condition for patients suffering from it, severely impacting their quality of life. The multiplicity of etiological factors and the uncertain pathogenesis make diagnosing this pathology challenging. Numerous hypotheses have been formulated, some more credible than others. One of the most widely accepted is that the rarefaction of the glycosaminoglycan layer, which covers the epithelium lining the bladder, is the main cause of IC/BPS [4]. In fact, the glycosaminoglycan layer serves as an initial barrier against urinary irritants. When this barrier is compromised or disrupted, irritants can penetrate and traverse into the submucosal layer, initiating inflammatory symptoms [20]. The underlying hypothesis for using intravesical hyaluronic acid instillations to treat interstitial cystitis posits that hyaluronic acid can restore the GAG layer on the bladder surface. By stimulating the production of new epithelial cells, hyaluronic acid helps to repair and reconstruct the bladder urothelium [21]. Our study demonstrated the efficacy of intravesical hyaluronic acid as a first-line treatment for IC/BPS. All three assessment questionnaires (ICSI, ICPI, and VAS) showed statistically significant results. Despite variations in the doses of hyaluronic acid used, the duration and frequency of therapy, and the length of follow-up, our findings are consistent with those reported in other studies in the literature. However, data from randomized controlled trials are lacking.

Numerous studies have employed ICSI, ICPI, and VAS scores to evaluate the efficacy of intravesical hyaluronic acid treatment of IC/BPS. Hung et al., Cervigni et al. and Lv YS et al. used ICSI, ICPI and VAS scores in patients with refractory IC/BPS who underwent a second-line intravesical hyaluronic acid treatment [22,23,24]. Giberti et al. also employed ICSI and ICPI scores to evaluate the efficacy of this treatment, but as a first-line treatment in patients with IC/BPS [25]. Riedl et al. analyzed the efficacy of intravesical hyaluronic acid as first-line therapy in patients diagnosed with IC/BPS and demonstrated a marked improvement in clinical symptoms as assessed by the VAS scale. In this study, intravesical instillations were administered weekly until patients showed significant improvement or a complete cessation of symptoms [26]. Akbay et al., Hung, M. J. et al. and Liang, C. et al. also confirm the efficacy of first-line therapy with hyaluronic acid in IC/BPS using ICSI, ICPI and VAS scores as a yardstick [27,28,29]. Thus, there are numerous studies in the literature demonstrating symptomatic efficacy in the treatment of both refractory or recurrent IC/BPS and as first-line treatment. Cervigni et al. and Lv YS et al. also evaluated the cystoscopic effects following this therapy and demonstrated a disappearance of submucosal vascular ectasia in most patients treated with intravesical hyaluronic acid [23,24]. It has also been shown that intravesical hyaluronic acid therapy in patients with IC/BPS has not only short-term but also long-term effects: in particular, Engelhardt et al. reported complete remission of bladder symptoms at a five-year follow-up, without the need for any additional therapy [30].

Our study, in addition to demonstrating the therapeutic efficacy of intravesical hyaluronic acid in IC/BPS, also showed that there was no statistically significant difference in terms of clinical outcome between patients who underwent ≤4 sessions and patients who underwent a higher number of sessions. This would seem to suggest that, for this type of treatment, more does not mean better. Similar clinical benefit is obtained with a number of sessions ≤4, so probably carrying out additional treatment steps is useless in terms of clinical benefit. Instead, it is probably more convenient to carry out fewer sessions in a short time (3–4 sessions in 6 months), and then carry out long-term maintenance sessions. Since this is one of the first studies to address this topic, these speculations will necessarily need further confirmation in future research.

The score obtained from each questionnaire was compared with the Minimally Important Clinical Difference (MICD) reported in the literature. The MICD represents the score difference that corresponds to a meaningful clinical improvement in patients. For the ICSI questionnaire, the MICD reported in the literature corresponds to a change of 5 points (sensitivity 81.3%, specificity 83.3%) [31]. In our study, the average difference reported was 7.12 points. As far as the ICPI questionnaire is concerned, the MICD reported in the literature corresponds to a difference of 3 points (sensitivity 84.4%, specificity 70.8%), which is significantly lower than the difference found in our study, which corresponds to 6.52 [30]. For the VAS, the clinically significant difference found in the literature corresponds to a difference of 3 points, once again less than 4.6, which is the difference reported by our analysis [32]. Therefore, we can conclude that the differences we observed (7.12 for ICSI, 6.52 for ICPI and 4.6 for VAS) are greater than the MICDs reported in the literature. Thus, the treatment not only resulted in statistically significant improvements, but also led to clinically significant enhancements.

The retrospective design of our study, while facilitating the analysis of real-world outcomes, limits our ability to infer causality between treatment and observed improvements. Among the limitations of the study, it is important to note that we used intravesical hyaluronic acid therapy as the sole treatment option, without combining it with any other possible treatments. Other limitations are represented by the lack of a placebo control group, the absence of adjusting variables, and the limited duration of follow-up.

## 5. Conclusions

Our study demonstrates that intravesical hyaluronic acid treatment as a first-line therapy for patients with IC/BPS leads to statistically and clinically significant improvements in symptoms, thereby enhancing the quality of life for these patients. We believe it is necessary to initiate multicenter randomized controlled trials involving larger patient cohorts and extended follow-up periods to corroborate our findings and refine therapeutic strategies for these patients, who suffer from a condition that is not yet fully understood. We reiterate that, although multiple therapeutic options effectively reduce the symptoms associated with IC/BPS, no treatment completely resolves the condition. Future studies are needed to explore new therapeutic horizons.

## Figures and Tables

**Table 1:** Baseline characteristics of patients.

Variables	
Number of patients	50
Age, median (IQR)	53.5 (47.0, 67.0)
Number of sessions, median (IQR)	4.0 (3.0, 5.0)
- Low (≤4)	31 (62%)
- High (>4)	19 (38%)

Values are expressed as IQR, ratio and absolute numbers. IQR: Interquartile range.

**Table 2:** Before- and after-treatment (6 months after the last administration of the drug) ICSI, ICPI and VAS results.

	Before Treatment	After Treatment
Interstitial Cystitis Symptom Index, median (IQR)	14.0 (10.0, 17.0)	5.5 (3.0, 8.0)
Interstitial Cystitis Problem Index, median (IQR)	13.0 (10.0, 15.0)	5.0 (2.0, 6.0)
Visual Analogue Scale, median (IQR)	9.0 (7.0, 10.0)	2.0 (1.0, 5.0)

Values are expressed as IQR and absolute numbers. IQR: Interquartile range.

**Table 3:** ICSI, ICPI and VAS pre- and post-treatment.

	Mean	SD	95% CI	*p*
**ICSI**				
ICSI pre	13.34	4.38	12.09–14.58	
ICSI post	6.22	4.36	4.97–7.46	
Difference	7.12	4.59	5.81–8.42	0.000
**ICPI**				
ICPI pre	11.90	3.54	10.89–12.90	
ICPI post	5.38	3.88	4.27–6.48	
Difference	6.52	4.20	5.32–7.71	0.000
**VAS**				
VAS pre	7.62	3.35	6.66–8.57	
VAS post	3.02	2.69	2.25–3.78	
Difference	4.60	3.31	3.65–5.54	0.000

ICSI: Interstitial Cystitis Symptoms Index, ICPI: Interstitial Cystitis Problem Index, VAS: Visual Analogue Scale.

**Table 4:** ICSI, ICPI and VAS post-treatment in low (≤4) versus high (>4) number of sessions.

	Mean	SD	95% CI	*p*
**ICSI**				
Low number of sessions	6.83	4.83	5.06–8.61	
High number of sessions	5.21	3.34	3.59–6.82	
Difference	1.62	-	−0.91–4.16	0.10
**ICPI**				
Low number of sessions	5.90	4.09	4.40–7-40	
High number of sessions	4.52	3.43	2.86–6.18	
Difference	1.38	-	−0.88–3.63	0.11
**VAS**				
Low number of sessions	3.16	2.84	2.11–4.20	
High number of sessions	2.78	2.48	1.59–3.98	
Difference	0.38	-	−1.21–1.96	0.32

ICSI: Interstitial Cystitis Symptoms Index, ICPI: Interstitial Cystitis Problem Index, VAS: Visual Analogue Scale.

## Data Availability

The raw data supporting the conclusions of this article will be made available by the authors, without undue reservation.

## References

[1] Doggweiler R., Whitmore K.E., Meijlink J.M., Drake M.J., Frawley H., Nordling J., Hanno P., Fraser M.O., Homma Y., Garrido G. (2017). A standard for terminology in chronic pelvic pain syndromes: A report from the chronic pelvic pain working group of the international continence society. Neurourol. Urodyn..

[2] Homma Y., Akiyama Y., Tomoe H., Furuta A., Ueda T., Maeda D., Lin A.T., Kuo H.-C., Lee M.-H., Oh S.-J. (2020). Clinical guidelines for interstitial cystitis/bladder pain syndrome. Int. J. Urol..

[3] Clemens J.Q., Meenan R.T., Rosetti M.C., Gao S.Y., Calhoun E.A. (2005). Prevalence and incidence of interstitial cystitis in a managed care population. J. Urol..

[4] Lazzeri M. (2006). The physiological function of the urothelium—More than a simple barrier. Urol. Int..

[5] Wyndaele J.J.J., Riedl C., Taneja R., Lovász S., Ueda T., Cervigni M. (2019). GAG replenishment therapy for bladder pain syndrome/interstitial cystitis. Neurourol. Urodyn..

[6] van de Merwe J.P., Nordling J., Bouchelouche P., Bouchelouche K., Cervigni M., Daha L.K., Elneil S., Fall M., Hohlbrugger G., Irwin P. (2008). Diagnostic criteria, classification, and nomenclature for painful bladder syndrome/interstitial cystitis: An ESSIC proposal. Eur. Urol..

[7] Butrick C.W., Howard F.M., Sand P.K. (2010). Diagnosis and treatment of interstitial cystitis/painful bladder syndrome: A review. J. Womens Health.

[8] Hanno P.M., Erickson D., Moldwin R., Faraday M.M., American Urological Association (2015). Diagnosis and treatment of interstitial cystitis/bladder pain syndrome: AUA guideline amendment. J. Urol..

[9] Grigoryan B., Kasyan G., Pivazyan L., Pushkar D. (2022). Pentosan polysulfate in patients with bladder pain syndrome/interstitial cystitis with Hunner’s lesions or glomerulations: Systematic review and meta-analysis. Ther. Adv. Urol..

[10] Panunzio A., Tafuri A., Mazzucato G., Cerrato C., Orlando R., Pagliarulo V., Antonelli A., Cerruto M.A. (2022). Botulinum Toxin-A Injection in Chronic Pelvic Pain Syndrome Treatment: A Systematic Review and Pooled Meta-Analysis. Toxins.

[11] Park J.J., Kim K.T., Lee E.J., Chun J., Lee S., Shim S.R., Kim J.H. (2024). Current updates relating to treatment for interstitial cystitis/bladder pain syndrome: Systematic review and network meta-analysis. BMC Urol..

[12] Mykoniatis I., Tsiakaras S., Samarinas M., Anastasiadis A., Symeonidis E.N., Sountoulides P. (2022). Monoclonal Antibody Therapy for the Treatment of Interstitial Cystitis. Biologics.

[13] Shen S.H., Peng L., Zeng X., Zhang J., Shen H., Luo D.Y. (2024). Intravesical Interferon Therapy vs Hyaluronic Acid for Pain Among Female Individuals with Interstitial Cystitis: A Randomized Clinical Trial. JAMA Netw Open..

[14] Ha T., Xu J.H. (2017). Interstitial cystitis intravesical therapy. Transl. Androl. Urol..

[15] Li J., Yi X., Ai J. (2022). Broaden Horizons: The Advancement of Interstitial Cystitis/Bladder Pain Syndrome. Int. J. Mol. Sci..

[16] Porru D., Campus G., Tudino D., Valdes E., Vespa A., Scarpa R.M., Usai E. (1997). Results of treatment of refractory interstitial cystitis with intravesical hyaluronic acid. Urol. Int..

[17] Nordling J., Jørgensen S., Kallestrup E. (2001). Cystistat for the treatment of interstitial cystitis: A 3-year follow-up study. Urology.

[18] Pyo J.S., Cho W.J. (2016). Systematic Review and Meta-Analysis of Intravesical Hyaluronic Acid and Hyaluronic Acid/Chondroitin Sulfate Instillation for Interstitial Cystitis/Painful Bladder Syndrome. Cell Physiol. Biochem..

[19] O’Leary M.P., Sant G.R., Fowler F.J., Whitmore K.E., Spolarich-Kroll J. (1997). The interstitial cystitis symptom index and problem index. Urology.

[20] Cervigni M. (2015). Interstitial cystitis/bladder pain syndrome and glycosaminoglycans replacement therapy. Transl. Androl. Urol..

[21] Abatangelo G., Vindigni V., Avruscio G., Pandis L., Brun P. (2020). Hyaluronic Acid: Redefining Its Role. Cells.

[22] Hung M.-J., Su T.-H., Lin Y.-H., Huang W.-C., Lin T.-Y., Hsu C.-S., Chuang F.-C., Tsai C.-P., Shen P.-S., Chen G.-D. (2014). Changes in sexual function of women with refractory interstitial cystitis/bladder pain syndrome after intravesical therapy with a hyaluronic acid solution. J. Sex. Med..

[23] Cervigni M., Natale F., Nasta L., Padoa A., Voi R.L., Porru D. (2008). A combined intravesical therapy with hyaluronic acid and chondroitin for refractory painful bladder syndrome/interstitial cystitis. Int. Urogynecol. J. Pelvic Floor Dysfunct..

[24] Lv Y.S., Zhou H.L., Mao H.P., Gao R., Wang Y.D., Xue X.Y. (2012). Intravesical hyaluronic acid and alkalinized lidocaine for the treatment of severe painful bladder syndrome/interstitial cystitis. Int. Urogynecol. J..

[25] Giberti C., Gallo F., Cortese P., Schenone M. (2013). Combined intravesical sodium hyaluronate/chondroitin sulfate therapy for interstitial cystitis/bladder pain syndrome: A prospective study. Ther. Adv. Urol..

[26] Riedl C.R., Engelhardt P.F., Daha K.L., Morakis N., Pflüger H. (2008). Hyaluronan treatment of interstitial cystitis/painful bladder syndrome. Int. Urogynecol. J. Pelvic Floor. Dysfunct..

[27] Akbay E., Çayan S., Kılınç C., Bozlu M., Tek M., Efesoy O. (2018). The short-term efficacy of intravesical instillation of hyaluronic acid treatment for bladder pain syndrome/interstitial cystitis. Turk. J. Urol..

[28] Hung M.J., Tsai C.P., Lin Y.H., Huang W.C., Chen G.D., Shen P.S. (2019). Hyaluronic acid improves pain symptoms more than bladder storage symptoms in women with interstitial cystitis. Taiwan. J. Obstet. Gynecol..

[29] Liang C.C., Lin Y.H., Hsieh W.C., Huang L. (2018). Urinary and psychological outcomes in women with interstitial cystitis/bladder pain syndrome following hyaluronic acid treatment. Taiwan. J. Obstet. Gynecol..

[30] Engelhardt P.F., Morakis N., Daha L.K., Esterbauer B., Riedl C.R. (2011). Long-term results of intravesical hyaluronan therapy in bladder pain syndrome/interstitial cystitis. Int. Urogynecol J..

[31] Yoshimura N., Uno T., Sasaki M., Ohinata A., Nawata S., Ueda T. (2022). The O’Leary-Sant Interstitial Cystitis Symptom Index is a clinically useful indicator of treatment outcome in patients with interstitial cystitis/bladder pain syndrome with Hunner lesions: A post hoc analysis of the Japanese phase III trial of KRP-116D, 50% dimethyl sulfoxide solution. Int. J. Urol..

[32] Abreu-Mendes P., Araújo-Silva B., Charrua A., Cruz F., Pinto R. (2022). Silodosin Improves Pain and Urinary Frequency in Bladder Pain Syndrome/Interstitial Cystitis Patients. J. Clin. Med..

